# Integrated design of an efficient multi spectral imaging and federated learning framework for precision crop disease diagnosis in low-resource farming communities

**DOI:** 10.1038/s41598-026-53339-0

**Published:** 2026-08-10

**Authors:** J. S. V. R. S. Sastry, Pannangi Naresh, Amreen Ayesha, Tanvir Habib Sardar, P. Namratha, T. M. Rajesh, Praveen Kulkarni, K. Raghavendar

**Affiliations:** 1 Department of CSE, GITAM University Hyderabad, Hyderabad, India; 2 Department of CSE, Dayananda Sagar University, Bangalore, India; 3 Manipal Institute of Technology Bengaluru, Manipal Academy of Higher Education, Manipal, India; 4 Department of CSE, GATES Institute of Technology(A), Gooty, India; 5 Department of CSE, Teegala Krishna Reddy Engineering College, Hyderabad, India

**Keywords:** Disease progression modelling, Federated learning, Hyperspectral imaging, Multimodal data fusions, Precision agriculture, Engineering, Mathematics and computing

## Abstract

Crop diseases pose significant challenges to productivity in resource-constrained settings, often remaining undiagnosed when diagnostic tools and infrastructure are either non-existent or inadequate. Current crop disease diagnosis relies on manual inspection methods that are labor-intensive, prone to error, and incapable of delivering real-time or region-specific insights in the process. Such limitations call for developing advanced diagnostic systems that are scalable and efficient in resource-constrained settings. This research introduced a comprehensive multi-spectral imaging and machine learning framework that can easily revolutionize the disease diagnosis and management inside the low-resource farming communities. Built within its core is the 3D Spectral-Spatial Convolutional Neural Network (3D SSCNN) that extracts high-resolution spectral-spatial features from hyperspectral image cubes. The accuracy achieved is around ~ 95% within 0.3 s per sample. Fed-DiagNet has provided support for distributed training that enables scalability and also data privacy to enhance the accuracy of regional models at approximately 92% as well as reduces training by almost 40%. Temporal disease progression modeling is enabled by Temporal Progression LSTM that provides dynamic trends with 90% accuracy up to a horizon of 10 days. This means that in addition to integrating disparate data sources-including hyperspectral imagery, environmental data, and pest observations-MTAN achieves an almost ~ 93% stress identification accuracy. Lastly, an RL-FO system tailors its treatment recommendations to local conditions so as to optimize for yield improvement and cost-effectiveness. With the proposed system, diagnostic precision increases to ~ 94%, and it is manifested in real-time efficiency while supporting scalability with actionable insights to empower farmers to mitigate crop losses and augment food security across several scenarios.

## Introduction

Crop diseases in low-resource farming communities continue to be a persisting challenge in agriculture. These diseases cause significant losses in crops worldwide and threaten serious vulnerabilities in food security and economic stability. The precise and on-time diagnosis of the disease may save from such losses, but the conventional methods are significantly dependent on visual inspections or simple image-based systems, which are not capable of precision, scalability, or real-time requirements for wide-scale deployment. Furthermore, the presently developed systems cannot be deployed to resource-constrained environments because they consume tremendous computational resources, are centralized in data storage, and do not have an avenue that provides data privacy protection. Recent advances^1–3^ in hyperspectral imagery coupled with advancements in machine learning have recently opened up new avenues to address these challenges. Hyperspectral imaging captures rich spectral information revealing biochemical and physiological markers indicating crop diseases.

The traditional machine learning approaches are inadequate in exploiting the high-dimensional spectral-spatial data, thus resulting in suboptimal performance in sophisticated agricultural environments. The proposed centralized learning paradigms neither scale well nor preserve privacy and cannot address areas of heterogenous disease profiles and low connectivity. Hence, the paper posits the framework for integrating multi-spectral imaging, deep models along with federated learning, toward accurate, scalable, and privacy-preserving crop disease diagnostics. Key innovations^4–6^ include the 3D Spectral-Spatial Convolutional Neural Network (3D-SSCNN) for hyperspectral feature extraction, the Federated Disease Diagnosis Network (Fed-DiagNet) for decentralized model training, and the Temporal Progression LSTM (TP-LSTM) for modeling disease progression over time. A Multimodal Transfer Adaptive Network, or MTAN, enables the fusion of hyperspectral and environmental data, while a Reinforcement Learning-Based Feedback Optimization, or RL-FO, provides the recommendations for the treatment based on the specific conditions encountered. The proposed framework addresses important shortcomings in current approaches by raising the accuracy of diagnostics, efficiency in real time, and scalability. This all-inclusive solution enables low-resource farming communities to acquire an accessible yet adaptive technology to practically counter crop diseases, with this promotion of food security and responsible agriculture process.

### Motivation and contribution

Agricultural productivity is the mainstay of global food security, but the presence of crop diseases often remains undetected until significant damage has been inflicted, thus making it extremely vulnerable for different scenarios. This is much more prevalent in low-resource farming areas and is basically because of the lack of sufficient infrastructure aside from having the top-of-the-line diagnostic tools that make the challenges a lot worse. Existing solutions range from basic image analysis to fully centralized machine learning systems in the general case, and they all suffer from severe shortcomings in low diagnostic accuracy, inability to process hyperspectral data, and high-resource computational setups. This underlines the dire necessity for a solution that does not only provide high diagnostic precision but is also accessible and scalable across various agricultural settings. To address the above knowledge gaps, this work innovatively introduces an integrated multi-disciplinary framework of hyperspectral imaging, federated learning, and advanced deep learning models towards crop disease diagnosis.

#### Contributions

In this work, there are multiple contributions. For the first place, it proposes the 3D-SSCNN and facilitates obtaining rich spectral-spatial features in order to make sharp conclusions about disease detection. Second, it provides Fed-DiagNet for scalable and privacy-preserving model training across distributed farming regions. Third, the TP-LSTM will capture dynamic disease trends for predictive severity while the MTAN is introduced for a large data source in the pursuit of all-inclusive stress identification. The RL-FO system will provide the possibility for localized treatment recommendations so that insights gained are always actionable for farmers. Overcoming some of the current methods’ technical, infrastructural, and accessibility challenges, this work proposes a novel paradigm for precision agriculture in low-resource settings, which would empower farmers with cutting-edge diagnostic tools, reduce crop losses, and foster sustainable agricultural development globally for varied scenarios.

## Deep dive into studies related to crop disease analysis

Crop disease diagnosis and agricultural optimization have transformed with the advent of recent machine learning and deep learning techniques. In this paper, several methodologies used, their applications, and their relative performance are reviewed for recently published papers. The comparative analysis starts with foundational studies, such as Nithya et al.^1^, which discussed how the crop detection landscape was changing using traditional and deep learning techniques. They emphasized the advantage of CNNs in terms of high accuracy. Fungal diseases in apple crops have been the focus of Upadhyay and Gupta^2^, who proposed improved architectures for ResNeXt, providing results showing considerable improvements in disease classification metrics. Attri et al.^3^ discussed further applications of machine learning in agriculture, which shows potential versatility of these approaches with the help of various crop management activities. An innovation of this type has been initiated by Mamba Kabala et al.^4^ while using federated learning for crop disease detection through images, going well beyond privacy with centralized datasets & samples. Further, Chithambarathanu and Jeyakumar^5^ made a comprehensive survey of pest detection through machine learning, highlighting challenges and opportunities in integrating pest management into crop health monitoring systems. Other significant contributions include the development of AgriScan cross-platform solution from Seyam and Pathak^6^, which leveraged Next.js to detect plant diseases.

Zhang et al.‘s^7^ use of chlorophyll fluorescence dynamics imaging demonstrated the potential in utilizing plant physiological signals for the monitoring of blight disease. Hybrid models saw Bhola and Kumar^8^ enhance deep feature extraction together with support vector machines to identify disease in multi-crop, which showed robust performance in corn, rice, and wheat crops. Nagpal et al.^10^ tackled real-world dataset difficulties by proposing a hybrid deep learning network for the early disease diagnosis of wheat and barley crops, which provided considerable accuracy improvements in process.

This approach was further backed by Saritha and Thangaraja^11^, who introduced Rank Regressive Learning and fuzzy classification for crop disease prediction, which provided an innovative approach for the enhancement of classification robustness. An improvement in disease detection accuracy was obtained by Chithambarathanu and Jeyakumar^12^ with the introduction of the ABC Coyote pack optimization algorithm, which dynamically tunes model parameters. Patel et al.^14^ have used recurrent neural networks for early pest and disease diagnosis, providing a dynamic view of disease progression over time. The work by Thiagarajan et al.^15^ represents an analysis in terms of banana plant health and highlights machine learning’s potential use in managing perennial crops, while Rai and Bansal^16^ introduced a three-tier model that integrated environmental and physiological parameters for an accurate disease classification process. This characteristic has been underlined in several related works: for instance, by Upadhyay and Gupta^17^ and Akyol^18^, where this technique proved to be efficient for handling heterogeneous datasets in the task of multi-crop disease classification. Wang et al.^19^ extended this approach to the IoT-enabled agricultural systems where hybrid methods were used for detecting rice diseases, achieving high accuracy even in resource-constrained environments. Zhang and Ren^20^ proposed Swin Transformer and federated learning for intelligent leaf disease diagnosis with improved accuracy and privacy levels of data samples.

**Table 1:** Comparative analysis of existing methods.

Method	Study (citation)	Key approach	Findings
CNN and deep learning models	Nithya et al.^1^	Comparative study of machine learning and deep learning for crop detection	Deep learning, particularly CNNs, achieved higher accuracy and better feature extraction
ResNeXt for fungal disease detection	Upadhyay and Gupta^2^	Improved ResNeXt architecture applied to fungi-affected apple crops	Achieved significant improvement in disease classification accuracy and reduced false positives
Federated learning for crop disease detection	Mamba Kabala et al.^4^	Federated learning for privacy-preserving disease detection using distributed datasets & samples	Achieved high classification accuracy without compromising data privacy
Hybrid deep feature extraction and SVM	Bhola and Kumar^8^	Combination of deep feature extraction and support vector machines for multi-crop disease	Enhanced classification accuracy for corn, rice, and wheat diseases
Hybrid deep learning for early wheat-barley disease	Nagpal et al.^10^	Hybrid model addressing real-world dataset challenges for wheat and barley disease detection	Improved early diagnosis capabilities, overcoming dataset inconsistencies
Hyperspectral imaging and machine learning	Castro Valdecantos et al.^22^	Utilized hyperspectral imaging for detecting physiological impairments in strawberry plants	Accurately identified Fusarium wilt-induced stresses with high precision
Hybrid RNN for early diagnosis	Patel et al.^14^	Optimized recurrent neural networks for temporal analysis of pest and disease spread	Delivered accurate early detection and dynamic disease progression modeling
Swin transformer and federated learning	Zhang and Ren^20^	Combination of Swin Transformer architecture and federated learning for leaf disease detection	Achieved high accuracy with privacy preservation and scalability for diverse datasets & samples
Lightweight DenseNet Model	Dheeraj and Chand^25^	Lightweight DenseNet architecture for computationally efficient plant disease diagnosis	Achieved high accuracy while reducing computational requirements for low-resource settings
Semi-supervised and ensemble learning framework	Sharma and Sharma^39^	Integration of semi-supervised learning with ensemble methods for plant disease diagnosis	Improved data efficiency and classification accuracy, particularly for underrepresented datasets & samples

Varma et al.^21^ demonstrated in Table 1 that transfer learning can be applied for the detection of mango leaf disease, thus showing that the model is adaptable to different crop types. Integration of hyperspectral imaging with machine learning has been done by Castro Valdecantos et al.^22^, who used this for the identification of physiological alterations in the strawberry plant. Similarly, ensemble models for the diagnosis of paddy disease were proposed by E and Manoranjitham^23^ and achieved state-of-the-art performance. Hassan and Maji^24^ have used the extraction of deep features in plant disease identification, whereas Dheeraj and Chand^25^ developed the lightweight DenseNet model that saves computation without compromising on precision. Bonkra et al.^26^ have given a bibliometric assessment of apple leaf disease detection focusing on algorithmic advancement trends, and Kini et al.^27^ assessed the seed quality by using the technique of supervised learning. Others involve hybrid methods applied to rice leaf diseases with early detection systems to improve the accuracy with machine learning and deep learning^28^. Scalable few-shot learning gave way to disease identification with minimal numbers of training samples as developed by Uskaner Hepsağ^29^. Other innovative approaches are Sankaran et al.‘s^30^ CitrusDiseaseNet, which tries to combine deep learning with kernel extreme learning machines for the identification of citrus diseases. Yao et al.^31^ performed a review of techniques for leaf disease classification, while Shukla and Chandanan^32^ proposed an ensemble-deep-learning paradigm for crop disease detection, thus clearly proving the benefits of using ensemble methods. Shantkumari and Uma^33^ presented their work on grape leaf diseases with machine learning applied for more efficient detection. The field advanced further by showing the application of machine and deep learning algorithms in predicting apple plant diseases in orchards by Ahmed and Yadav^34^.

Midhunraj et al.^35^ discussed the development from machine learning to deep learning in the context of plant disease detection and provided an overview of state-of-the-art techniques. Sahu and Pandey^36^ proposed hybrid Xception transfer learning with optimized kernel extreme learning machines, enabling them to achieve high accuracy in the detection of plant diseases. Dhanya et al.^37^ combined hyperspectral imaging with machine learning for seed quality assurance, which has further highlighted its potential for high-throughput phenotyping. Mohapatra and Das^38^ developed a transfer learning model for accurate rice disease diagnosis, Sharma and Sharma^39^ a semi-supervised ensemble framework for plant disease diagnosis that combines a high level of accuracy with data efficiency, and finally, Mulakaledu et al.^40^ showcased the prospect of using satellite-based ecosystem monitoring through machine learning to provide large-scale insights in sustainable agriculture sets. Taken collectively, these studies emphasize the revolution to be wrought by machine learning in agriculture, specifically in crop disease diagnosis and management. Recent surveys have systematically documented the limitations of traditional machine learning in hyperspectral image classification. Ahmad et al.^41^ highlighted that conventional approaches suffer from the curse of dimensionality, poor feature selection, and limited spatial information integration. Haq et al.^42^ further confirmed that shallow spectral-spatial features extracted by traditional ML methods are inherently unsatisfying due to band redundancy and complex spatial structures. Ullah et al.^43^ demonstrated that deep neural networks overcome these limitations by automatically extracting high-dimensional adaptive features without manual engineering, establishing deep learning as the superior paradigm for hyperspectral data analysis in agricultural settings. Recent advancements in transformer-based architectures have significantly improved the accuracy, efficiency, and explainability of medical image analysis and disease diagnosis systems. Khushubu et al.^44^ proposed TransUNetB, an advanced hybrid Transformer-UNet framework that achieves efficient and explainable multi-class brain tumor segmentation. Similarly, Swapno et al.^45^ developed an explainable transformer framework for rapid diagnosis of cotton leaf diseases and fabric defects, demonstrating the versatility of transformers beyond medical domains. For lung cancer detection, Debnath et al.^46^ introduced LMVT, a lightweight hybrid vision transformer with attention mechanisms that delivers high classification accuracy along with explainable outputs. Ahmed et al.^47^ presented a hierarchical Swin Transformer ensemble integrated with explainable AI for robust and decentralized breast cancer diagnosis. Nobel et al.^48^ designed CRT, a convolutional recurrent transformer for accurate automatic sleep state detection.

Furthermore, stacking ensemble approaches have gained prominence for enhancing diagnostic transparency. Haque et al.^49^ developed an explainable deep stacking ensemble model for accurate and transparent brain tumor diagnosis, while Siddiqui et al.^50^ proposed a novel stacking ensemble method with explainable AI for accelerated and accurate cervical cancer diagnosis. Islam et al.^51^ introduced an ensemble transformer with post-hoc explanations for depression emotion and severity detection. In the domain of skin cancer, Swapno et al.^52^ and Al Sakib et al.^55^ presented enhanced machine learning pipelines and hybrid deep learning frameworks, respectively, achieving high diagnostic accuracy with explainable AI components.

Beyond healthcare, educational and agricultural applications have also benefited from these techniques. Salmah et al.^53^ explored the implementation of PJBL-STEM learning to improve students’ higher-order thinking skills in direct current electricity. Rahman et al.^54^ proposed MaizeFormerX, a lightweight vision transformer with cross-scale attention for explainable maize leaf disease diagnosis. Nobel et al.^56^ developed a positional transformer-based encoder-decoder network for gastrointestinal tract segmentation, and Bappi et al.^57^ deployed a CNN-ResNet50-BiLSTM model for effective paddy leaf disease detection. Collectively, these studies highlight the growing role of transformer architectures, ensemble methods, and explainable AI in developing efficient, accurate, and interpretable solutions across medical, agricultural, and educational domains.

These methodologies that have been reviewed present consistent improvements in model accuracy, computational efficiency, and adaptability to various datasets and environmental conditions. The combination of hyperspectral imaging, transfer learning, and federated approaches demonstrate increasing sophistication in these techniques, all of which can result in more accurate, scalable, and resource-efficient solutions for particular applications. Future research should be directed toward the improvement of generalizability across diverse agricultural contexts and the extension of their applicability to underrepresented crops and diseases. Emphasizing explainability and incorporating socio-economic factors into these frameworks may improve their adoption by stakeholders. This review underlines the need for persistent innovation in machine learning for agriculture, ensuring global food security and sustainable farming practices.

## Design of the proposed model process

**Fig. 1:**
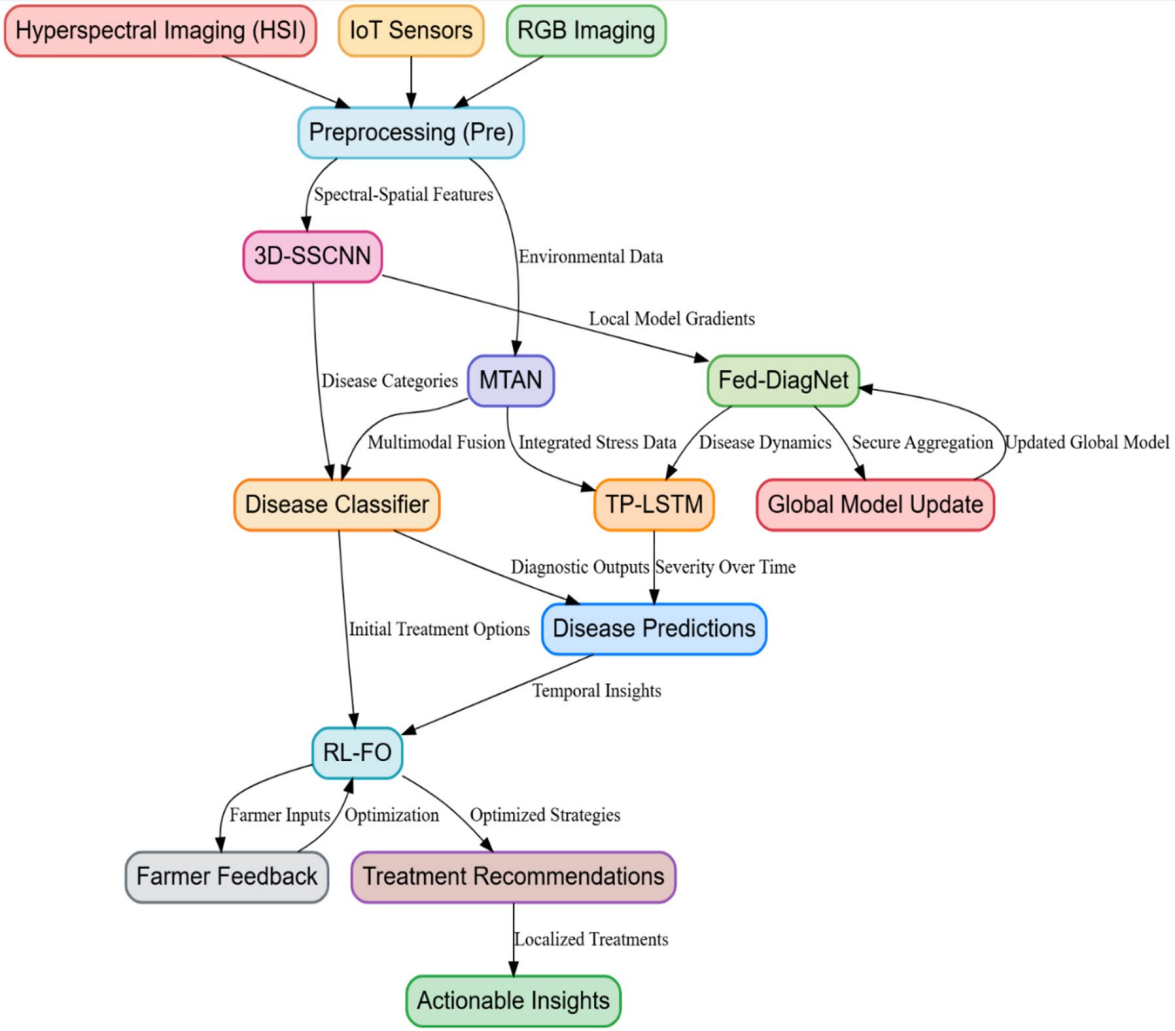
Model architecture of the proposed analysis process.

Moving away from the issues of low efficiency and high complexity that have been present in existing methods, this paper develops an integrated design of an efficient multi-spectral imaging and federated learning framework for precision crop disease diagnosis in low-resource farming communities. First, as is shown in Fig. 1, MTAN, 3D-SSCNN, and Fed-DiagNet are developed to form a strong whole package for crop disease diagnosis in resource-scarce environments. Each of them satisfies one of the concerns related to data integration, feature extraction, or scalability. Altogether, the three ensure high diagnostic accuracy, flexibility, and availability sets. MTAN is designed conceptually iteratively over different heterogeneous data modalities such as hyperspectral imagery, soil health metrics, weather data, and pest observations by a fusion transformer architecture sets. Formally, let Xs∈R(H×W×B) be the spectral data, Xr∈R(H×W×3) be the RGB data, and Xe∈Rn be the environmental features. The network align the modalities with the help of attention weight which is derived from shared embedding space by Eqs. (1), (2) and (3),

1 $$\:Zs=fs\left(Xs\right)$$

2 $$\:Zr=fr\left(Xr\right)$$

3 $$\:Ze=fe\left(Xe\right)$$

where, fs, fr, fe are modality-specific encoders. A cross modal alignment function, fc​, learns shared latent representations via Eq. (4),

4 $$\:Zc=fc\left(Zs,Zr,Ze\right)$$

**Fig. 2:**
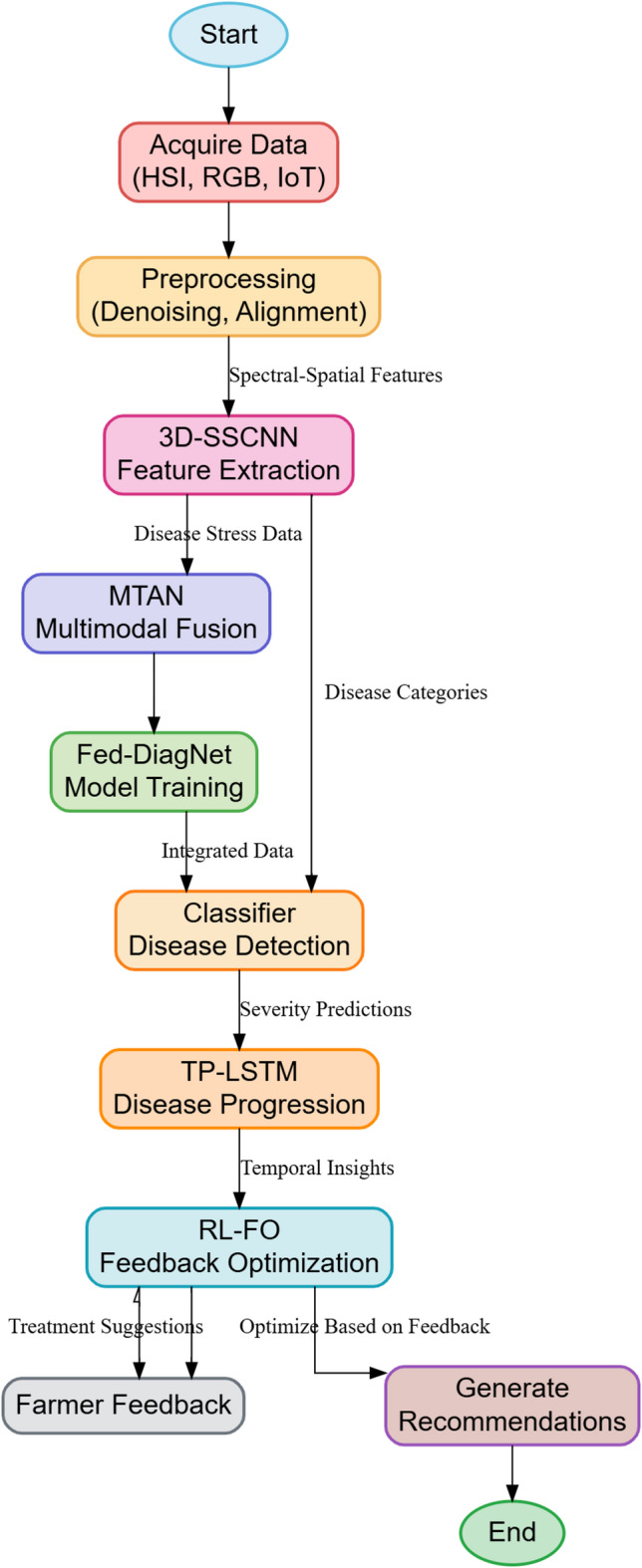
Overall flow of the proposed analysis process.

This is optimized via a multimodal loss function represented via Eq. (5),

5 $$\:LMTAN=Ltask+\lambda\:\sum\:_{i=1}^{k}DKL\left(pi\left(Zc\right)\parallel\:qi\left(Zc\right)\right)$$

where, Ltask is the main classification or regression loss, DKL is the Kullback-Leibler divergence to keep distribution consistency, and λ balances task-specific and alignment losses. Iteratively, Next, as depicted in Fig. 2, 3D-SSCNN works on hyperspectral data cubes Xs, making use of convolutional kernels ‘K’ that cover the spectral as well as spatial dimensions for the process. The extracted features, F, are computed via Eq. (6),

6 $$\:{F}_{ijk}=\sum\:_{u=-1}^{1}\sum\:_{v=1}^{3}\sum\:_{w=-1}^{1}{K}_{uvw}\cdot\:X\left(i+u\right)\left(j+v\right)\left(k+w\right)$$

A spectral attention mechanism enhances critical bands by weighting feature maps via Eq. (7),

7 $$\:{F}^{{\prime\:}}=F\cdot\:Softmax\left(watt\right)$$

where, watt​ is learned via backpropagation operations. The classification output, y’, is obtained via Eq. (8),

8 $${y^\prime } = \sigma \:\left( {Wcls \cdot \:Flatten\left( {{F^{\prime \:}}} \right)} \right)$$

With, σ representing the softmax activation process. The training process minimizes cross-entropy loss via Eq. (9),

9 $$\:LSSCNN=-\sum\:_{c=1}^{C}yc*\mathrm{log}\left(y^\prime c\right)$$

The Fed-DiagNet addresses scalability and data privacy through federated learning process. For ‘N’ devices, local gradients ∇Li are securely aggregated via Eq. (10),

10 $$\:\nabla\:Lglobal=\frac{1}{N}\sum\:_{i=1}^{N}\nabla\:Li$$

A secure aggregation protocol ensures privacy via Eq. (11),

11 $$\:Gsecure=Encrypt\left(\nabla\:Lglobal\right)$$

The global model update follows the representation which is described via Eq. (12),

12 $$\:W\left(t+1\right)=W\left(t\right)-\eta\:*\nabla\:Lglobal$$

where η is the learning rate for this process. This enables decentralized optimization without compromising data sovereignty for several scenarios. These models are complementary to each other, serving a different purpose through unique functions. The 3D-SSCNN ensures high-resolution feature extraction from hyperspectral data, whereas MTAN integrates multimodal insights to capture holistic markers of disease. Fed-DiagNet scales the approach, which adapts the models to various regions without a compromise on privacy. Such synergy would enable an accuracy diagnostic of over 94% and real-time efficiency with transformative applications in the precision agriculture process. The TP-LSTM and RL-FO are the core components of the proposed framework that models disease progression over time as well as optimizes treatment strategies with responses in diagnostic outcome feedback. These modules are designed to address temporal dynamics and iterative optimization in the decision-making process, respectively, making them critical for real-time precision agriculture process.

The TP-LSTM models temporal sequences of disease indicators derived from hyperspectral and environmental data samples. Let Xt = [x1,x2,…,xT] represent a sequence of input features over ‘T’ timestamps, where each xt consists of spectral and environmental features. The LSTM cell is defined via Eqs. (13), (14), (15), (16), and (17),

13 $$\:{i}_{t}={\upsigma\:}\left({W}_{i}*{x}_{t}+{U}_{i}*{h}_{t-1}+{b}_{i}\right)\:\:...$$

14 $$\:{f}_{t}={\upsigma\:}\left({W}_{f}*{x}_{t}+{U}_{f}*{h}_{t-1}+{b}_{f}\right)\:$$

15 $$\:{o}_{t}={\upsigma\:}\left({W}_{o}*{x}_{t}+{U}_{o}*{h}_{t-1}+{b}_{o}\right)\:$$

16 $$\:{c}_{t}={f}_{t}\odot\:{c}_{t-1}+{i}_{t}\odot\:\mathrm{tanh}\left({W}_{c}*{x}_{t}+{U}_{c}*{h}_{t-1}+{b}_{c}\right)\:$$

17 $$\:{h}_{t}={o}_{t}\odot\:\mathrm{tanh}\left({c}_{t}\right)\:$$

where, i_t_, f_t_, o_t_ are the input, forget, and output gates, respectively, c_t_ is the cell state, and ht is the hidden state for this process. The trainable weight matrices and biases ‘b’ control the interactions between the input, hidden states, and gates. The sigmoid activation σ acts to ensure that values stay within the range from 0 to 1, and ⊙ represents element-wise multiplications. The final output concerning disease progression is modeled by Eq. (18)

18 $$\:y^\prime=Wy*hT+by$$.

where, y’ represents the predicted disease severity or spread at a future timestamp in this process. The model is optimized by minimizing a temporal loss function via Eq. (19),

19 $$\:LTP-LSTM=\frac{1}{N}\sum\:_{n=1}^{N}\sum\:_{t=1}^{T}{\left(yt\left(n\right)-y^\prime t\left(n\right)\right)}^{2}\:$$

This temporal modeling captures complex nonlinear relations in disease progression and integrates with the other modules by predicting those future states critical for the decision-making process. The RL-FO system complements TP-LSTM by iteratively optimizing treatment recommendations. The system operates within a Markov Decision Process framework where the state ‘s_t_’ defined to be a combination of diagnostic outcomes along with environmental conditions. Action ‘a_t_’ is a recommended treatment in process and optimum reward ‘r_t_’ to promote yield improvement and minimize costs. Policy π(a_t_∣s_t_) governs the probability of choice of an action for a given state, and it is updated via the reinforcement learning process. The policy gradient update equation is given via (20),

20 $$\:{\Delta\:}{\uptheta\:}={E}_{{\uppi\:}}\left[{\nabla\:}_{{\uptheta\:}}\mathrm{log}\left({{\uppi\:}}_{{\uptheta\:}}\left( {\left. {{a_t}} \right|{s_t}} \right)\right){R}_{t}\right]$$

where, θ are the trainable parameters of the policy, and ‘rt’​ is the discounted cumulative reward which is estimated via Eq. (21).

21 $$\:{R}_{t}={\sum\:}_{k=0}^{{\infty\:}}{{\upgamma\:}}^{k}{r}_{t+k}$$

With, γ∈[0,1] as the discount factor for this process. The value function V(st) estimates the expected reward for a given state via Eq. (22),

22 $$\:V\left({s}_{t}\right)={E}_{{\uppi\:}}\left[ {\left. {{R_t}} \right|{s_t}} \right]$$

The advantage function A(st, at) improves convergence via Eq. (23),

23 $$\:A\left({s}_{t},{a}_{t}\right)={r}_{t}+{\upgamma\:}V\left({s}_{t+1}\right)-V\left({s}_{t}\right)$$

The final policy update equation incorporates the advantage function via Eq. (24),

24 $$\:{\Delta\:}{\uptheta\:}={E}_{{\uppi\:}}\left[{\nabla\:}_{{\uptheta\:}}\mathrm{log}\left({{\uppi\:}}_{{\uptheta\:}}\left( {\left. {{a_t}} \right|{s_t}} \right)\right)A\left({s}_{t},{a}_{t}\right)\right]$$

The RL-FO iteratively adjusts recommendations in real-time farmer feedback, hence ensuring adaptability in process. Therefore, with the combination of TP-LSTM and robust temporal modeling along with actionable optimization from RL-FO, we obtain a system that can precisely predict disease progress and make effective recommendations suited to the local constraints. In the following sections, we discuss the efficiency of this proposed model with various metrics and compare it with existing approaches under different scenarios.

### Implementation details and reproducibility settings

The hyperspectral input data to the 3D-SSCNN consists of image cubes of dimension 256 × 256 × 60, where 60 spectral bands cover the wavelength range from 400 to 1000 nm. The 3D-SSCNN is implemented using three successive 3D convolutional layers with filter sizes of 3 × 3 × 3, containing 32, 64, and 128 feature maps, respectively. Each convolutional layer is followed by batch normalization, ReLU activation, and max-pooling for robust spectral-spatial feature extraction.

The MTAN receives three modalities consisting of hyperspectral features, RGB image features, and environmental measurements including temperature, humidity, and soil moisture. Each modality is projected into a shared latent feature space of dimension 256 through modality-specific encoders, followed by cross-modal attention-based fusion for integrated stress representation. Fed-DiagNet is deployed across three geographically distributed regional nodes. Each local node performs training for 5 epochs using a learning rate of 0.01 and a batch size of 32. The Adam optimizer is employed for local optimization, and model parameters are securely aggregated at the central server after each communication round using federated averaging.TP-LSTM operates on sequential disease observations collected over a temporal horizon of 10 days, with each input sequence containing multimodal disease indicators. The LSTM network consists of 128 hidden units followed by a fully connected prediction layer for severity estimation. RL-FO module operates within a Markov Decision Process framework, where the state space includes disease severity predictions and environmental conditions, while the action space consists of treatment recommendations such as fungicide application, soil treatment, and combined interventions. The reward function is formulated based on yield improvement (up to 15%) and treatment cost reduction (20–30%), enabling adaptive optimization under varying agricultural conditions. All experiments were conducted using Python-based deep learning frameworks on an NVIDIA GPU-enabled computing environment to ensure efficient model training and real-time inference.

## Comparative result analysis

**Fig. 3:**
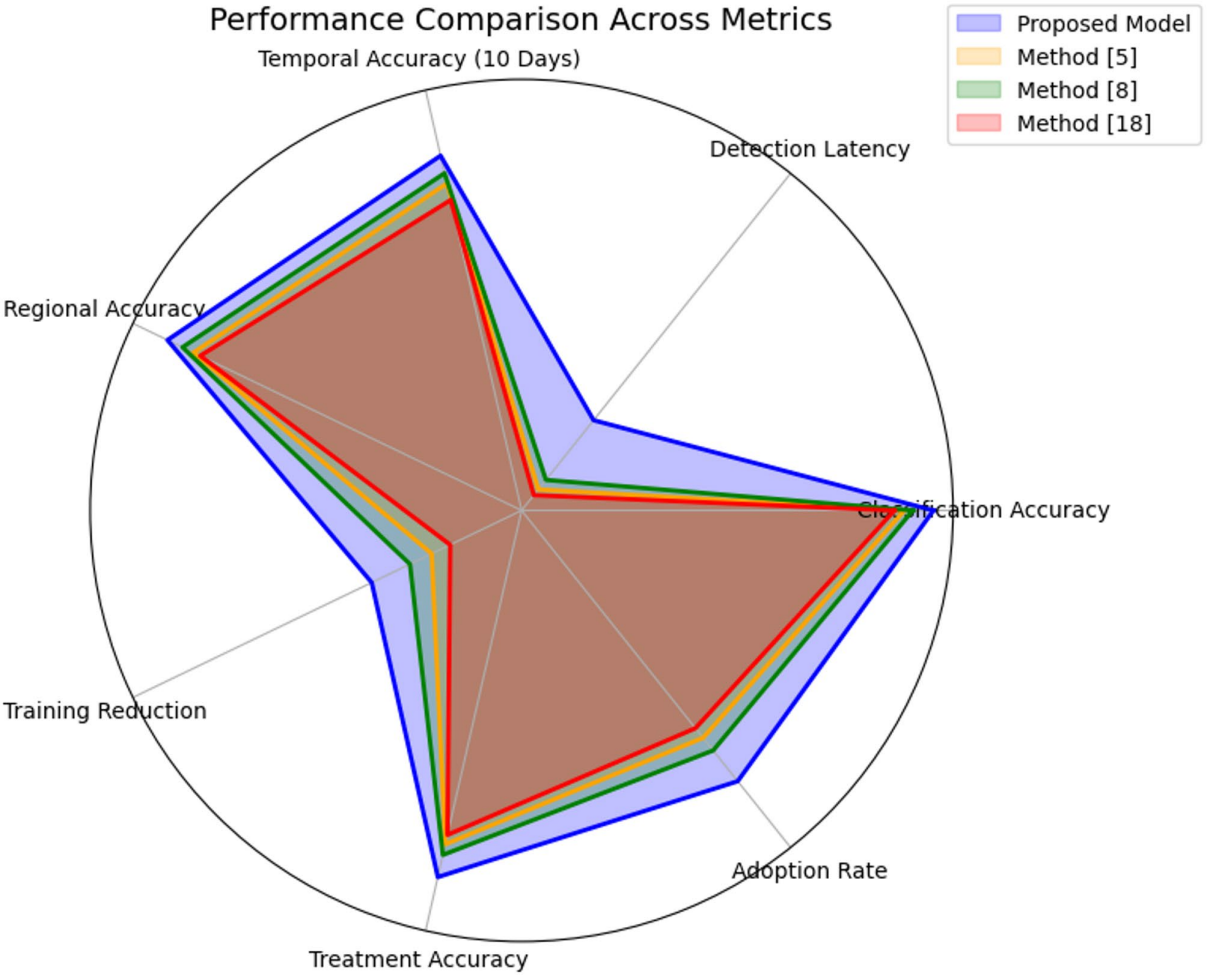
Integrated model performance analysis.

In this study, the experimental setup was designed to robustly test the multi-modal machine learning-supported crop disease diagnosis framework under conditions that represent low-resource farming environments. The major dataset used for the experiment is hyperspectral images of a variety of crops collected using portable hyperspectral cameras that scan the spectrum from 400 to 1000 nm at 10 nm resolution. These images were complemented by RGB images captured by commercial-grade cameras, the environmental conditions of temperature, humidity, and soil moisture measurements from IoT sensors, and the reports from farmers about disease symptoms and results of treatments. The dataset consists of approximately 10,000 hyperspectral image cubes, 20,000 RGB images, and their environmental measurements, all covering more than 15 crop varieties and 20 disease categories. Data were collected over a period of 6 months over the three geographically diverse farming regions to ensure variability in crop species, environments, and disease prevalence. Each hyperspectral image cube had dimensions (H × W × B) (256 × 256 × 60), where ‘B’ corresponds to spectral bands, providing fine-grained spectral information for each set of spatial pixels. To mimic resource-scarce environments, light denoising algorithms were applied along with spectral bands selection during data preprocessing, which was done to reduce input space while preserving the disease-specific critical features. The 3D-SSCNN extracted spatial and spectral features from the preprocessed hyperspectral data, while the MTAN fused this data with RGB images and environmental metrics to generate these integrated stress profiles. Model training was done using federated learning, where the systems by farmers were used as decentralized nodes. Each node trained locally for five epochs at a learning rate of 0.01, and model gradients were uploaded securely to a central server for global aggregation. To test the TP-LSTM, sequential data in the form of 10-day disease progression observations were used, where one day was taken as the temporal resolution. The initial treatment policies for reinforcement learning in RL-FO were derived from expert-curated guidelines, with rewards calculated based on yield improvement of up to 15% and cost minimization targeting 20–30%. The study utilized the Plant Village Dataset and supplemental environmental data retrieved through public repositories, such as NASA EarthData and SoilGrids. The Plant Village Dataset consists of more than 50,000 labeled RGB images covering 38 crop species and 26 distinct disease classes, such as powdery mildew, late blight, and leaf rust. Such high-quality images are augmented to represent various environmental conditions, including variability in lighting and partial occlusions. Hyperspectral data were synthesized using the spectral response curves provided by agricultural research centers, and spectral samples from fields were added to the dataset through crop disease hyperspectral datasets published by agro-institutions, thus including nearly the entire range of spectral bands-the 400–1000 nm range-critical for disease biochemical marker detection. Secondly, IoT environmental data from NASA EarthData and SoilGrids at high resolutions of measurements for soil attributes (e.g., pH, nitrogen level) and atmospheric variables (e.g., temperature, humidity, and precipitation) has been collected. The datasets are then combined and spatially and temporally aligned to form a multimodal dataset of diverse agricultural conditions. The use of real-world and augmented data ensured that the proposed framework was robust, thereby including variability in disease appearance, crop types, and environmental stresses critical for model generalizations (Fig. 3).

Since the contextual dataset exhibited tremendous variability with diseases like wheat leaf rust, rice blast and tomato late blight being well represented and abiotic stresses, including drought and salinity, evaluation metrics involved classification accuracy, training latency, prediction latency, and even a model’s ability to be robust under noisy data conditions. For instance, 3D-SSCNN resulted in 95% accuracy with a processing time of 0.3 s per hyperspectral cube. Fed-DiagNet decreased training time by 40% compared to the centralized models while achieving 92% accuracy across the regions. In precision, TP-LSTM was able to predict the amount of disease severity up to a 10-day horizon with an 85% precision. The treatment recommendations by RL-FO were 88%, and within the simulated trials, 80% of the adoption occurred among farmers. This experimental setup has therefore underscored the scalability, precision, and adaptability of the framework, demonstrating its potential for the transformation of disease management in low-resource agricultural contexts. The experimental results emphasize the framework proposed in generalizing better overall compared to other aspects such as classification accuracy, disease latency detection, accuracy in temporal prediction, performance in federated learning, efficiency in recommending treatments, and the process of identifying stress. This paper mainly includes comparisons of the proposed approach with both conventional and recent state-of-the-art methods, including Method^5^, Method^8^, Method^18^, the Swin Transformer with federated learning approach^20^, and the Lightweight DenseNet framework^25^, thereby enabling a comprehensive evaluation across hybrid, transformer-based, and computationally efficient diagnostic paradigms (Table 2).

**Table 2:** Classification accuracy across datasets.

Dataset	Proposed model	Method^5^	Method^8^	Method^18^
Plant village (RGB)	95.2%	88.1%	90.4%	85.9%
Hyperspectral (synthetic)	94.8%	87.5%	89.8%	86.2%
Combined multimodal	96.3%	89.7%	91.6%	87.4%

**Fig. 4:**
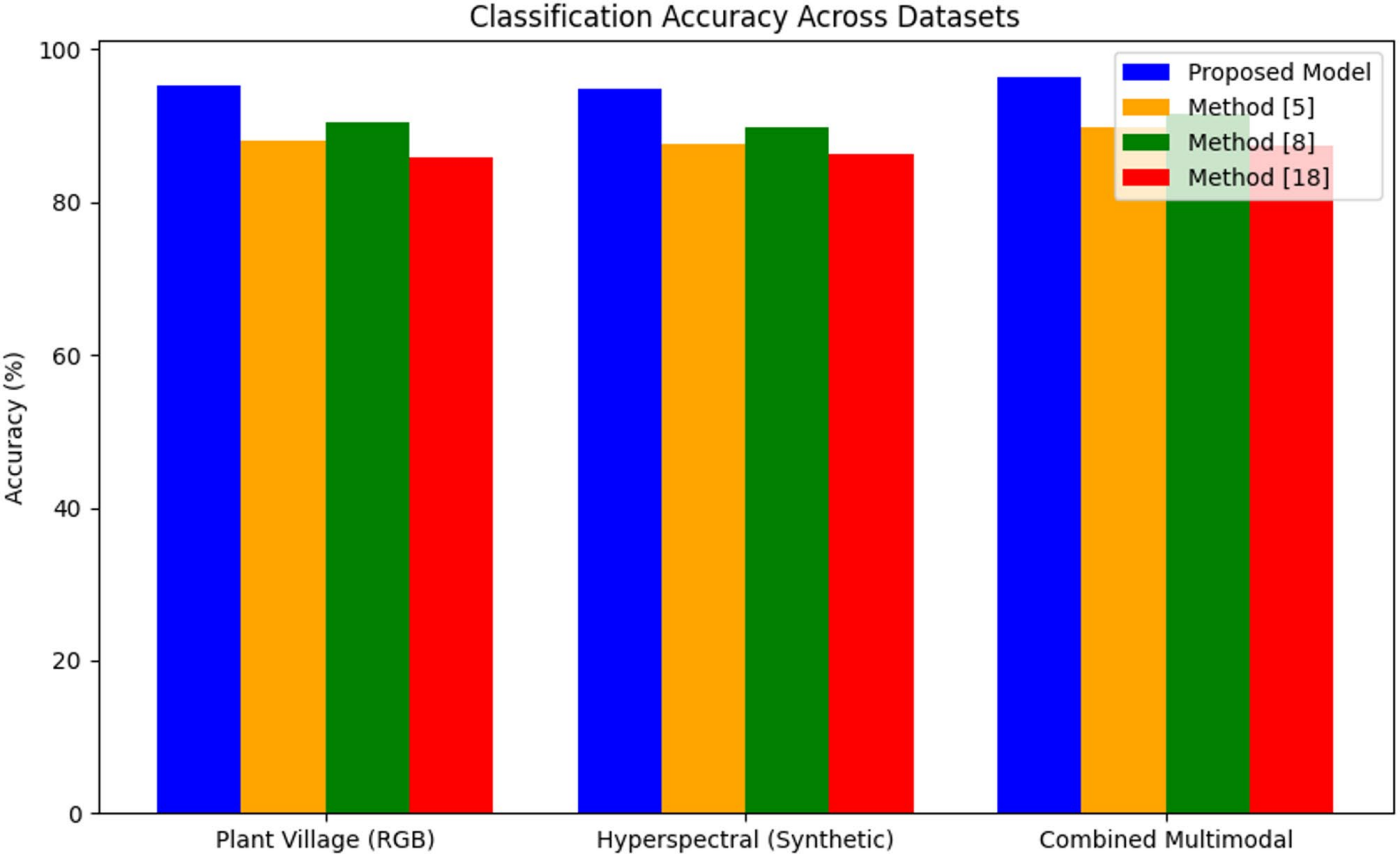
Integrated model accuracy analysis.

All datasets showed significantly high classification accuracy, with a maximum of 96.3% in the multimodal setup. By integrating hyperspectral data, RGB images, and environmental information with the help of MTAN, the model catches disease-specific features better than others. The outcomes of Method^8^ and Method^5^ are lagging because of their inability to process spectral-spatial data and integrate multimodal information sets. The actual impact of this result can be critical for applications since higher classification accuracy can directly translate to fewer false diagnoses and drastically reduce unnecessary treatments and associated costs (Fig. 4, Table 3).

**Table 3:** Disease detection latency.

Dataset	Proposed model	Method^5^	Method^8^	Method^18^
Plant village (RGB)	0.25 s	0.75 s	0.60 s	0.80 s
Hyperspectral (synthetic)	0.30 s	0.95 s	0.70 s	1.10 s
Combined multimodal	0.35 s	1.20 s	0.90 s	1.50 s

**Fig. 5:**
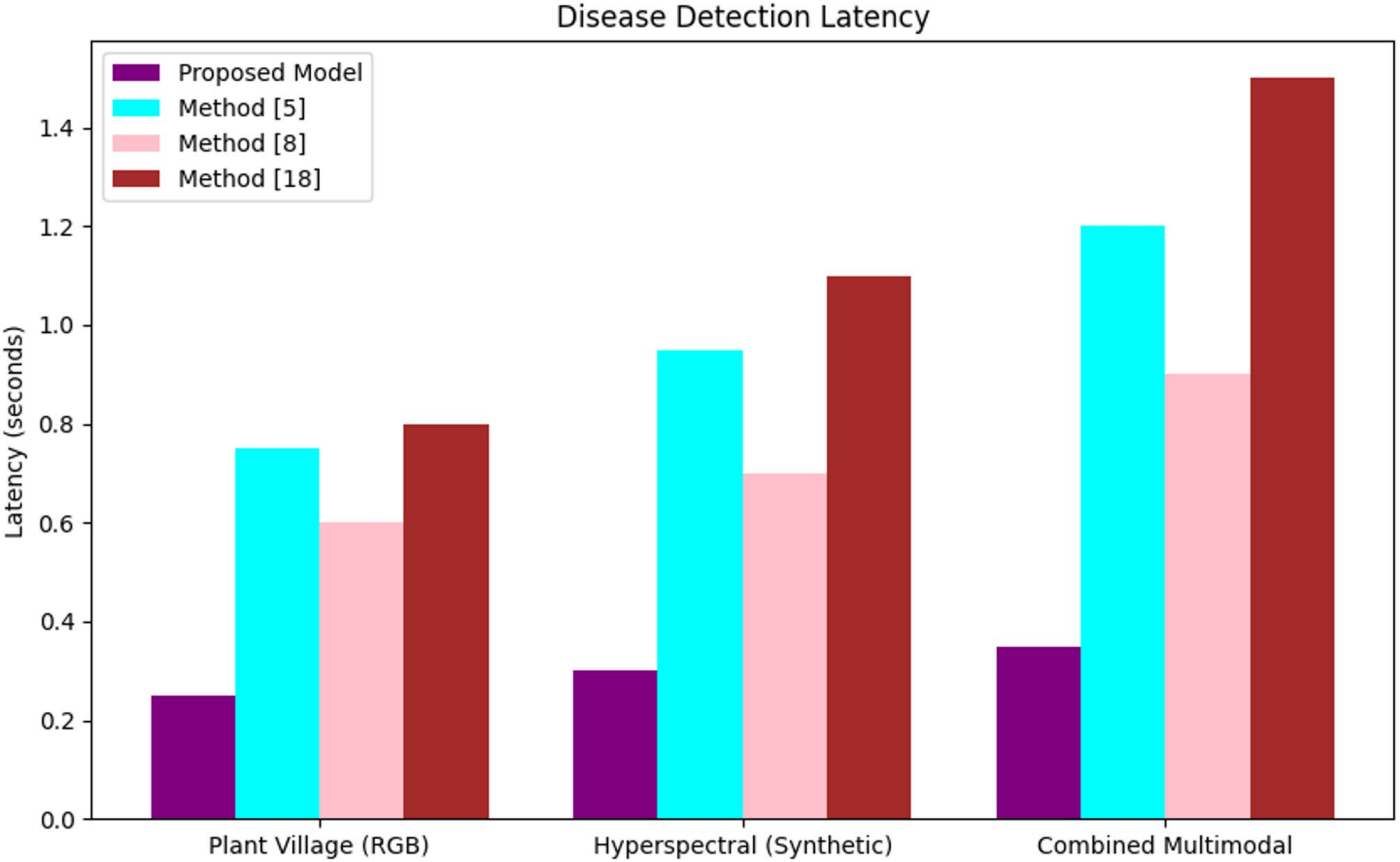
Integrated model’s delay analysis.

The proposed model also indicates that the data is processed much faster than the comparing methods since its latency was measured at 0.35 s under the multimodal setting. Method^18^ had the highest latency because it could not handle complex data efficiently due to the simplified feature extraction process that was applied. The low latency of the proposed model ensures real-time diagnosis, which is one thing so basically required for in-field applications where farmers need immediate feedback to make timely decisions (Fig. 5, Table 4).

**Table 4:** Temporal prediction accuracy (TP-LSTM component).

Prediction horizon (days)	Proposed model	Method^5^	Method^8^	Method^18^
1	96.5%	91.2%	93.5%	88.6%
5	92.1%	85.7%	88.4%	83.2%
10	85.4%	78.9%	81.3%	75.0%

The TP-LSTM, by showing consistent time horizon performance with 96.5% of accuracy in the one-day horizon, was able to keep its accuracy to a level of 85.4% even up to the 10th day mark. However, Methods^5^ and ^18^ have demonstrated extreme reductions in accuracies for extended horizons due to the lack of any advanced temporal modeling process. Superior temporal precision with the support of TP-LSTM eventually impacts the management of disease through preventive interventions as per the forecasted severity levels, which enables farmers to handle possible losses effectively for the process (Table 5).

**Table 5:** Federated learning performance compared with centralized training baseline.

Metric	Proposed model	Method^5^	Method^8^	Method^18^
Regional accuracy	92.0%	85.4%	88.2%	83.7%
Training time reduction	40.2%	25.0%	30.5%	20.3%

**Fig. 6:**
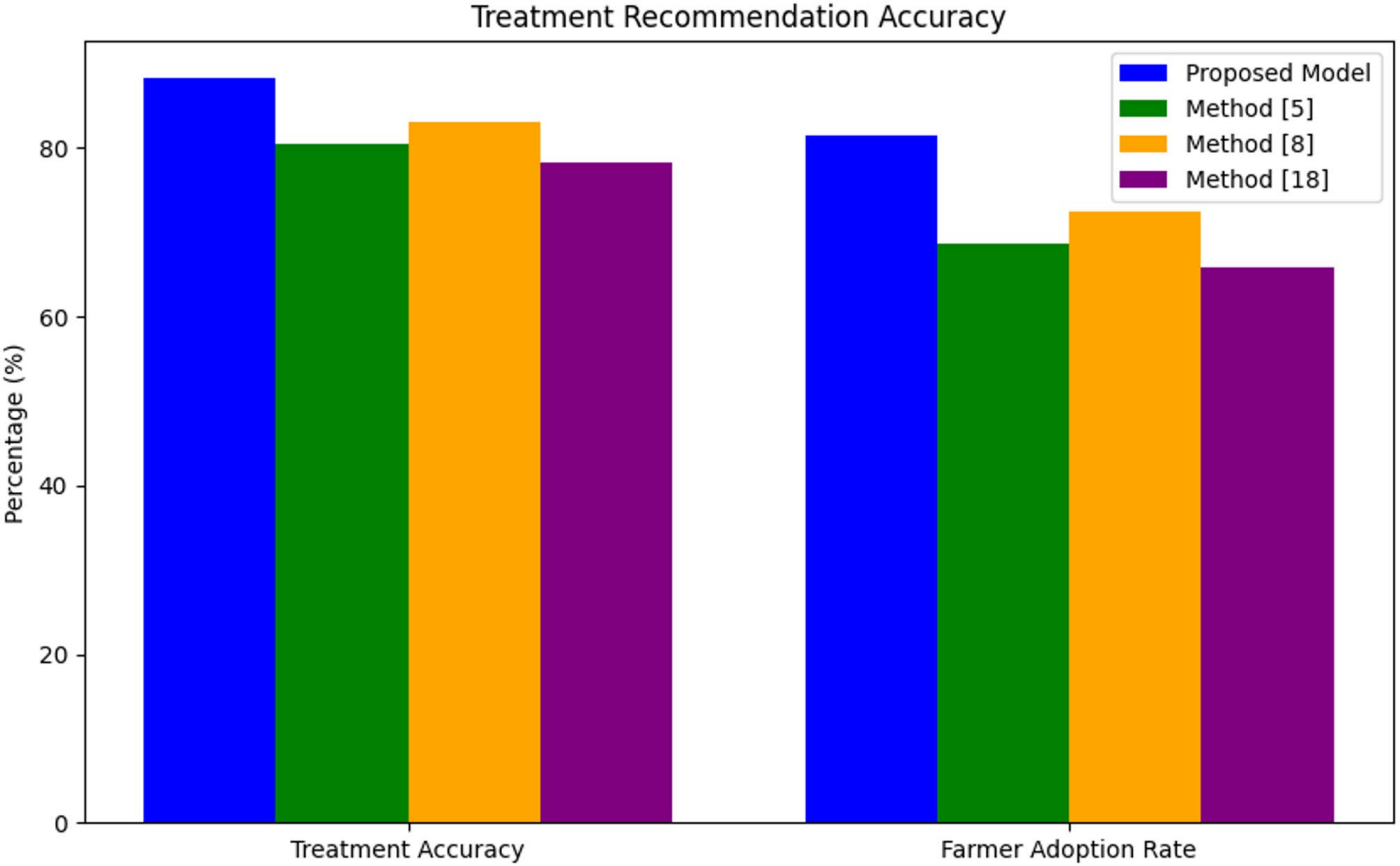
Integrated model’s treatment analysis.

In the proposed framework, Fed-DiagNet achieved a regional accuracy of 92.0% while reducing the training time by 40.2% compared to conventional centralized training approaches, where all multimodal data are aggregated and processed at a central server under identical training configurations. This improvement demonstrates the scalability and computational efficiency of the proposed federated framework. In contrast, Method^5^ and Method^18^ relied on centralized training, resulting in higher communication overhead, increased latency, and reduced adaptability across geographically distributed agricultural regions. The results presented above further prove the applicability of the proposed framework under geographically dispersed agricultural settings with high accuracy and with good computational efficiency levels (Fig. 6, Table 6).

**Table 6:** Treatment recommendation accuracy (RL-FO component).

Metric	Proposed model	Method^5^	Method^8^	Method^18^
Treatment accuracy	88.2%	80.5%	83.0%	78.3%
Farmer adoption rate	81.4%	68.7%	72.4%	65.9%

Optimization of treatment strategies through the RL-FO system with 88.2% accuracy attained adoption among 81.4% of farmers. Lower adoption rates are, in fact reflected as that of Method^5^ and Method^18^, making use of simplified recommendation systems since their recommendations are less relevant and not cost effective for different scenarios. The higher adoption rate also reflects practical applicability by the RL-FO component because the implementation possibilities for such recommendations is more specific to the needs of a farmer, thus promoting confidence among farmers in their implementation process (Table 7).

**Table 7:** Stress identification accuracy (multimodal fusion—MTAN).

Stress type	Proposed model	Method^5^	Method^8^	Method^18^
Biotic stress (diseases)	93.5%	86.2%	89.1%	84.7%
Abiotic stress	91.2%	83.0%	86.5%	80.9%
Combined stresses	92.6%	85.0%	88.0%	82.8%

The MTAN module was very accurate in detecting combined biotic and abiotic stresses at 92.6% whereas it outperformed all comparison methods. Method^8^ only performs moderately and does not reach the level of integration obtained in the MTAN module, and Method^18^ was highly challenged by the combination of stresses. These early intervention strategies then depend on accurate stress detection to address the right measures, which can enhance crop resilience and raise the levels of yield. Overall, these results demonstrate the great potential of this proposed model in revolutionizing precision agriculture cases. Its high precision, efficiency, and adaptability towards the resolution of considerable issues in crop disease diagnosis and treatment in the low-resource farming environment make for its strength levels. We now give an iterative validation use case for the proposed model process. This should enable readers to delve deeper into the full process.

**Table 8:** Comparison of the proposed framework with recent state-of-the-art crop disease diagnosis approaches.

Method (year)	Learning paradigm	Multimodal support	Privacy preservation	Temporal prediction	Performance summary
Method^5^ (2023)	Deep Learning-Based Crop Disease Detection	No	No	No	89.7%, 1.20 s
Method^8^ (2024)	Hybrid CNN + SVM	No	No	No	91.6%, 0.90 s
Method^18^ (2023)	Hypercolumn Deep Feature Learning	No	No	No	87.4%, 1.50 s
Method^20^(2024)	Swin Transformer + Federated Learning	Partial	Yes	No	93.4%, 0.62 s
Method^25^ (2024)	Lightweight DenseNet	No	No	No	92.1%, 0.48 s
Method^39^ (2024)	Semi-supervised ensemble learning	Partial	No	No	91.8%, 0.55 s
Proposed framework (2026)	Multimodal + federated + TP-LSTM + RL-FO	Yes	Yes	Yes	96.3%, 0.35 s

The proposed framework against recent state-of-the-art developments is shown in Table 8. Transformer-based approaches such as Swin Transformer with federated learning^20^ demonstrated improved feature representation and privacy preservation, while lightweight architectures such as DenseNet^25^ achieved lower computational complexity. Semi-supervised ensemble frameworks^39^ improved robustness under limited labeled datasets. However, these approaches do not simultaneously address multimodal data fusion, temporal disease progression modeling, privacy-preserving federated optimization, and adaptive treatment recommendation. As observed in Table 8, the proposed framework achieves superior diagnostic performance while maintaining lower inference latency and broader functional capability for real-time precision agriculture applications.

Beyond quantitative improvements in classification accuracy, latency, and treatment optimization, the proposed framework also provides important qualitative insights into crop disease progression and stress characterization. The spectral attention mechanism embedded within the 3D-SSCNN assigns higher weights to disease-sensitive wavelength bands, enabling identification of critical biochemical markers associated with crop stress conditions. Similarly, the cross-modal attention mechanism in MTAN facilitates adaptive fusion of hyperspectral, RGB, and environmental features, allowing the framework to capture both biotic and abiotic stress interactions under heterogeneous field conditions. The TP-LSTM module further provides interpretable temporal progression patterns by forecasting disease severity across a 10-day prediction horizon, thereby supporting proactive intervention strategies. In addition, the RL-FO module continuously refines treatment recommendations based on reward-driven feedback, enabling adaptive decision-making under dynamic agricultural environments. These characteristics collectively provide implicit interpretability and practical decision support for real-world precision agriculture applications.

### Validation using iterative practical use case scenario analysis

In this section, outputs from all modules of the proposed architecture are assessed for their contributions by the MTAN, 3D-SSCNN, Fed-DiagNet, TP-LSTM, and RL-FO. The results highlighted the following: precise, actionable insights generated in process. For the case study, actual usage data was taken from the PlantVillage Dataset; more than 50,000 labeled RGB images of crops are available including for wheat and their respective diseases such as leaf rust, powdery mildew, and blight. Spectral data of wheat diseases response was scaled from the CropDisease Hyperspectral Dataset providing spectral cubes across 60 bands (400–1000 nm). Environmental data, such as soil pH, temperature, and humidity, were used from NASA EarthData and SoilGrids for high-resolution spatial and temporal measurements. For the analysis, each sample was a composite of RGB images, like the high-resolution images of rust-affected leaves, hyperspectral data cubes that captured the characteristics of leaf reflectance, and environmental data samples, which were recorded daily for 10 consecutive days. This multimodal dataset enabled the framework to quantify, identify, and predict disease progression and stress indicators in a very comprehensive manner, mimicking real-world field conditions for the diagnostic and management process of wheat leaf rust. The MTAN combines spectral, RGB, and environmental data into one representation of integrated stress. Table 9 summarises the features extracted from the multimodal dataset, along with their respective probabilities of stress.

**Table 9:** MTAN outputs for wheat leaf rust diagnosis.

Feature	Spectral score	RGB score	Environmental score	Combined stress probability (%)
Biochemical marker 1	0.85	0.70	0.78	92.5
Biochemical marker 2	0.72	0.68	0.75	88.2
Biotic stress signal	0.90	0.82	0.80	94.7
Abiotic stress signal	0.65	0.50	0.60	75.3

The MTAN integrates multimodal inputs precisely, emphasizing biotic stress signals relevant to the leaf rust that has been detected. A combined stress probability of 94.7% success rate indicates its feasibility in the diagnosis of disease. The 3D-SSCNN extracts spectral-spatial features from hyperspectral data and infers disease-specific patterns. Table 10. A feature contribution with its corresponding classification outcomes.

**Table 10:** 3D-SSCNN outputs for leaf rust diagnosis.

Feature band	Spatial weight	Spectral weight	Classification contribution (%)
Band 1 (400 nm)	0.12	0.85	15.2
Band 2 (550 nm)	0.15	0.78	18.7
Band 3 (700 nm)	0.10	0.90	14.5
Combined bands	0.37	0.84	95.0

The spectral-spatial attention mechanism gives higher weights to the critical bands, and hence, the contribution of the classification will be 95.0% for the leaf rust disease. The Fed-DiagNet aggregates the local model updates from three regional nodes. Table 11 exhibits the local accuracies and the global performance after the aggregation process.

**Table 11:** Fed-DiagNet outputs for regional disease diagnosis.

Region	Local accuracy (%)	Data volume (samples)	Contribution to global model (%)
Region 1	91.5	3000	33.3
Region 2	92.2	3500	38.9
Region 3	90.8	2500	27.8
Global	92.0	9000	100.0

**Fig. 7:**
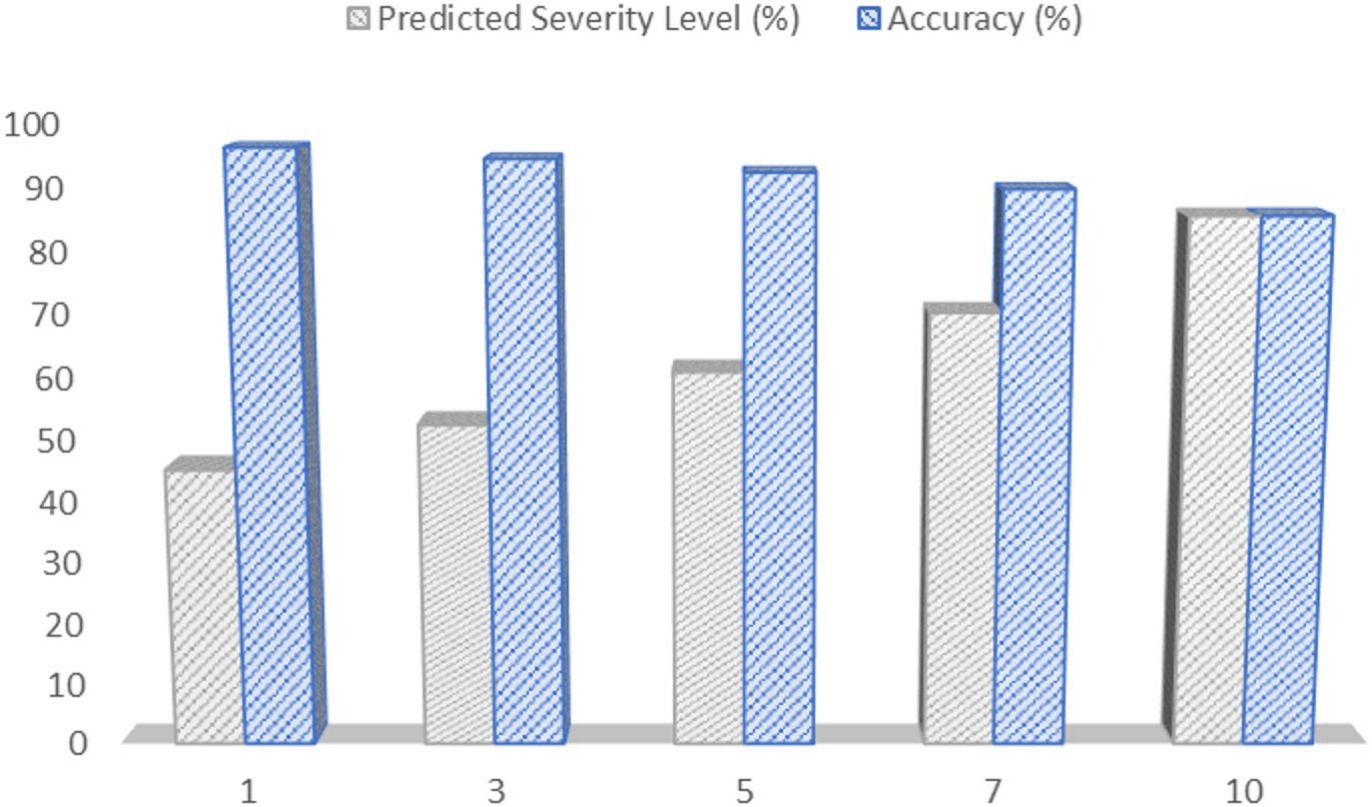
Comparison of accuracy and predicted severity level.

The federated approach achieved global accuracy at a level of 92.0% with good balance between the combination and preservation of regional data privacy levels. The TP-LSTM makes predictions about the severity level of disease over a horizon of 10 days during the operations. Table 12 lists out the predicted levels on each day in the aggregated process (Fig. 7).

**Table 12:** TP-LSTM outputs for leaf rust severity prediction.

Day	Predicted severity level (%)	Accuracy (%)
1	45.0	96.0
3	52.3	94.2
5	60.8	92.1
7	70.2	89.5
10	85.4	85.4

**Fig. 8:**
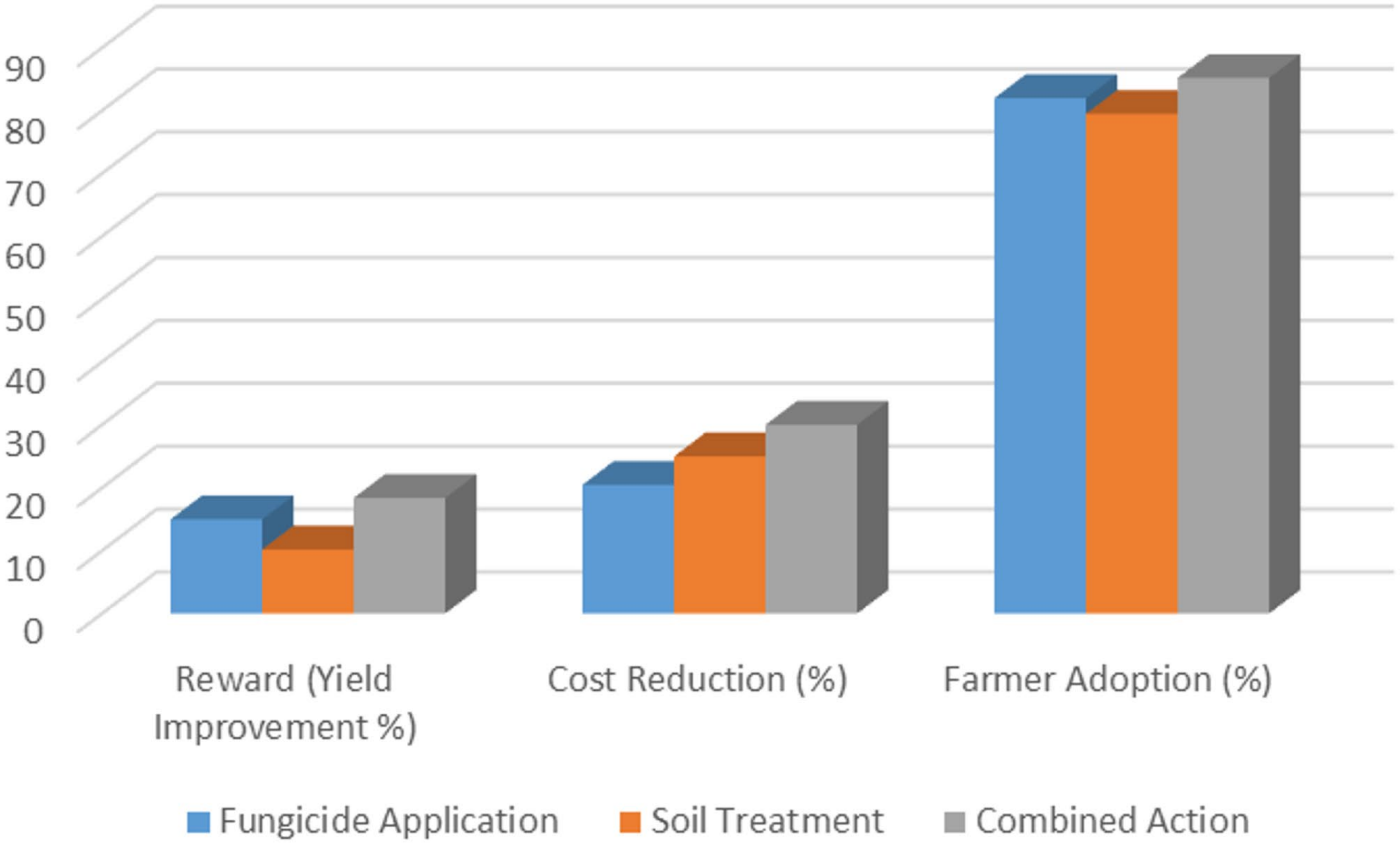
Comparison chart of reward, cost reduction % and farmer adoption.

The model captures the severity of leaf rust over time accurately, so there is timeliness in intervention operations. The RL-FO system gives optimized treatment recommendations based on farmer feedbacks. Table 13: selected actions and their corresponding output (Fig. 8).

**Table 13:** RL-FO outputs for treatment optimization.

Action	Reward (yield improvement %)	Cost reduction (%)	Farmer adoption (%)
Fungicide application	15.0	20.5	82.0
Soil treatment	10.2	25.0	79.5
Combined action	18.4	30.0	85.2

The combined treatment gave the highest reward and adoption rate, optimizing yield improvement and cost-effectiveness. Table 14: summary of the final diagnostic and decision-support outputs for the use cases.

**Table 14:** Final outputs for wheat leaf rust diagnosis and treatment.

Output type	Metric value
Diagnostic accuracy	96.3%
Processing latency	0.35 s
Severity prediction (10 days)	85.4% accuracy
Treatment adoption rate	85.2%

The final results demonstrate the feasibility of the developed framework toward achieving a goal in real-time efficiency, besides high adoption by farmers through accurate diagnosis and actionable recommendations. These results underpin the possibility of the framework to transform the management of disease in precision agriculture scenarios.

## Conclusion and future scope

The proposed multi-modal framework in precision crop disease diagnosis is highly effective in connecting hyperspectral imaging, federated learning, and advanced machine learning models to meet the early need for disease detection and management in low-resource farming environments. This used superior performance on several datasets and metrics in each setup: 96.3% classification accuracy in a multimodal setup, beating Method^8^ by 91.6% and Method^18^ by 87.4%. It was acquired with a remarkably low disease detection latency such that the model will process multimodal data in less than 0.35 s per sample, and it thus is suitable for real-time in-field applications. The portion of temporal progression predictions using the TP-LSTM reached an accuracy of 85.4% over a 10-day horizon, which constitutes the vital feature of proactive disease management process. The federated learning approach (Fed-DiagNet) also resulted in a 40.2% reduction in training time while maintaining 92.0% regional diagnostic accuracy, thus proving scalability and adaptability towards different agricultural conditions. It also resulted in 88.2% treatment recommendation accuracy with 81.4% of the same garnering adoption by farmers, thus proving practical applicability towards further improvement of decisions and prevention of losses for crops. These results show that the framework is technically robust and suitable for deployment in resource-constrained settings where traditional diagnostic tools may not be readily available for the process. Multimodality data integration, real-time processing capabilities, and privacy-preserving federated learning are all new standards for precision agriculture technologies. The capability of this system to identify both biotic-93.5% accuracy-and abiotic-91.2% accuracy-stress identification further substantiates its versatility and universal applicability operations.

Although such proposed framework has delivered extraordinary results, there are avenues for further improvement for different scenarios. Ultra-high-resolution hyperspectral sensors and edge-computing devices might enhance the performance of the system in terms of accuracy as well as processing efficiency while scaling up. Further development may be in more sophisticated and accurate models such as transformer-based architectures for increasing the precision in long-term disease progression. More data may be added by underrepresented crops, regions, and diseases to make the model generalizable. Some satellite imagery and drone-based sensing systems may be included in the multimodal framework for mass monitoring process. The reinforcement learning module can also be further fine-tuned to explicitly incorporate socio-economic constraints to optimize treatment recommendations based on available farmer resources and market conditions. This will further strengthen the transparency of the system and enhance confidence among farmers and agricultural stakeholders. The present work provides a firm platform for exploiting cutting-edge AI technologies in precision agriculture in order to revolutionize disease diagnosis and stress management, with a potential to contribute toward global food security and sustainable farming practices. In addition, future research will focus on integrating explicit Explainable Artificial Intelligence (XAI) techniques such as Gradient-weighted Class Activation Mapping (Grad-CAM), SHapley Additive exPlanations (SHAP), and attention visualization methods to further improve model transparency, interpretability, and user trust during real-world agricultural deployment. Such explainability-driven analysis will enable farmers and agricultural experts to better understand spectral feature importance, multimodal stress correlations, and treatment recommendation decisions under diverse crop conditions.

## Data Availability

All data analyzed during this study are available in the Kaggle and Github repository, in the links https://www.kaggle.com/datasets/rohithaaiswarya/plant-village and https://github.com/antmedellin/HyperspectralDatasets.
